# Mercury Induces an Unopposed Inflammatory Response in Human Peripheral Blood Mononuclear Cells *in Vitro*

**DOI:** 10.1289/ehp.0900855

**Published:** 2009-08-19

**Authors:** Renee M. Gardner, Jennifer F. Nyland, Sean L. Evans, Susie B. Wang, Kathleen M. Doyle, Ciprian M. Crainiceanu, Ellen K. Silbergeld

**Affiliations:** 1 Department of Environmental Health Sciences, Johns Hopkins Bloomberg School of Public Health, Baltimore, Maryland, USA; 2 Department of Pathology, Microbiology, and Immunology, University of South Carolina School of Medicine, Columbia, South Carolina, USA; 3 Department of Molecular Microbiology and Immunology and; 4 Department of Biostatistics, Johns Hopkins Bloomberg School of Public Health, Baltimore, Maryland, USA

**Keywords:** immunotoxicity, inflammation, mercury, multilevel modeling

## Abstract

**Background:**

The human immune response to mercury is not well characterized despite the body of evidence that suggests that Hg can modulate immune responses, including the induction of autoimmune disease in some mouse models. Dysregulation of cytokine signaling appears to play an important role in the etiology of Hg-induced autoimmunity in animal models.

**Objectives:**

In this study, we systematically investigated the human immune response to Hg *in vitro* in terms of cytokine release.

**Methods:**

Human peripheral blood mononuclear cells (PBMCs) were isolated from 20 volunteers who donated blood six separate times. PBMCs were cultured with lipopolysaccharide and concentrations of mercuric chloride (HgCl_2_) up to 200 nM. Seven cytokines representing important pathways in physiologic and pathologic immune responses were measured in supernatants. We used multilevel models to account for the intrinsic clustering in the cytokine data due to experimental design.

**Results:**

We found a consistent increase in the release of the proinflammatory cytokines interleukin-1β (IL-1β) and tumor necrosis factor-α, and concurrent decrease in release of the antiinflammatory cytokines interleukin 1-receptor antagonist (IL-1Ra) and IL-10 in human PBMCs treated with subcytotoxic concentrations of HgCl_2_. IL-4, IL-17, and interferon-γ increased in a concentration–response manner. These results were replicated in a second, independently recruited population of 20 different volunteers.

**Conclusions:**

Low concentrations of HgCl_2_ affect immune function in human cells by dysregulation of cytokine signaling pathways, with the potential to influence diverse health outcomes such as susceptibility to infectious disease or risk of autoimmunity.

As demonstrated in animal models, mercury affects immune function in a complex manner that depends on both the species of Hg used and the genetic background against which exposure takes place. In genetically susceptible mouse strains, inorganic Hg (iHg) and organic Hg species induce autoimmunity, resulting in a lupuslike condition (Havarinasab and Hultman 2005). Coexposure to the antigenic stimulus lipopolysaccharide (LPS), a potent activator of the innate immune system, can shift susceptibility to the immunotoxic effects of iHg such that nonsusceptible mouse strains become susceptible and susceptible strains experience an exacerbation of iHg-induced autoimmune disease (Abedi-Valugerdi et al. 2005).

Mouse models of Hg-induced autoimmune disease suggest that cytokine regulation is an important determinant in terms of pathophysiologic outcome (Bagenstose et al. 1999; Haggqvist and Hultman 2005; Hu et al. 1999). Particular attention has been paid to the balance of cytokines produced by the T-helper T_H_1 and T_H_2 subsets, and dysregulation of cytokine release is involved in driving responses of autoreactive T cells toward the development of autoimmunity (Bagenstose et al. 1998; Haggqvist and Hultman 2003).

Humans may also be susceptible to the immunotoxic effects of Hg. Hg exposure has been associated with increased risk of lupus and greater severity of scleroderma (Arnett et al. 1996; Cooper et al. 2004). Environmental and occupational exposures to Hg compounds are correlated with serum levels of autoantibodies, a pathology commonly found in murine models of Hg-induced autoimmunity (Alves et al. 2006; Silbergeld et al. 2005; Silva et al. 2004). However, other studies have failed to find a correlation between occupational Hg exposure and markers of immune dysfunction (Barregard et al. 1997; Ellingsen et al. 2000).

Human immune function is highly variable in terms of both response to infection and conditions such as autoimmune disease, with differences between sexes and among people of different genetic backgrounds (Fairweather et al. 2008; Hill 2006; Klein 2000). It is plausible that the immunotoxic response to Hg will also be highly variable across people as a result of genetic or environmental factors.

The goal of our two-phase study was to characterize and quantify the human immune response to iHg *in vitro*, in terms of both the magnitude of changes in cytokine release and the individual variability observed in cytokine release. In choosing the Hg species to use, we selected iHg because it has been demonstrated to be the most potent inducer of immunotoxicity in animal models (Havarinasab and Hultman 2005). In addition, methylmercury is metabolized to and retained in the body as iHg (Hg^2+^) (Suda et al. 1992; Vahter et al. 1994) and is likely the Hg species that eventually acts upon immune cells after exposure to methylmercury (Havarinasab et al. 2007). In phase 1 of this study, we used a multilevel study design in which volunteers (level 1) were asked to return for multiple visits (level 2) for blood donations so that we could assess both between- and within-individual variability in cytokine response (level 3). In phase 2, we tested the predictive capability of the models we developed in phase 1 to characterize the immune response to iHg *in vitro* using peripheral blood mononuclear cells (PBMCs) from another independently selected group. We also collected information about individual blood donors in order to characterize the effects of these variables on cytokine response *in vitro*.

## Materials and Methods

### Tissue culture chemicals and reagents

All chemicals were obtained from Sigma-Aldrich (St. Louis, MO) unless otherwise noted. Phosphate-buffered saline (PBS), penicillin-streptomycin, and l-glutamine were obtained from Mediatech (Manassas, VA). RPMI 1640 and heat-inactivated fetal bovine serum (hiFBS) were obtained from Invitrogen (Carlsbad, CA). LPS was reconstituted in sterile PBS as a 400-ng/mL stock solution and frozen at −20°C in aliquots; all experiments used freshly thawed LPS aliquots from the same batch. All plastics used were certified endotoxin-free by the manufacturer. Ficoll-Paque Plus (GE Healthcare, Piscataway, NJ) had endotoxin levels of < 0.12 endotoxin units (EU)/mL, PBS had < 0.005 EU/mL, and culture media (before LPS treatment) had < 0.01 EU/mL.

### Human subjects

In phase 1, a convenience sample of 20 healthy adult volunteers (10 males and 10 females) were recruited from the Johns Hopkins Medical Institutions community. Volunteers were required to be between 18 and 40 years of age. To avoid obvious sources of variability within the immune system, our exclusion criteria included personal or immediate family history of autoimmune disease, use of steroidal medications (including birth control pills), regular use of nonsteroidal antiinflammatory drugs (NSAIDs), receipt of organ transplant, and pregnancy. Volunteers were asked to donate 20 mL of blood and to answer a brief questionnaire about their lifestyle and health status. Volunteers were asked to repeat this process six times with at least 1 month separating each visit [see Supplemental Materials, Figure 1, available online (doi:10.1289/ehp.0900855.S1)]. All volunteers were required to be free of illness (to the best of their knowledge) at the time of the blood draw. Blood collection for all volunteers occurred over a total period of 18 months.

In phase 2, an additional 20 healthy adult volunteers (10 males and 10 females) were recruited from the same community. These volunteers, who met the same inclusion and exclusion criteria as those recruited during phase 1, were asked to donate blood and answer a questionnaire only once.

All volunteers gave written informed consent before participation in this study. All activities were conducted in accordance with U.S. regulations and were approved by the Johns Hopkins Bloomberg School of Public Health Committee on Human Research. At no point were any volunteers exposed to Hg as a result of participation in this study.

### Blood collection and cell culture

Venous blood (20 mL) was collected by a trained phlebotomist under aseptic conditions into sodium heparin-coated Vacutainer tubes (Becton Dickinson, Franklin Lakes, NJ). Whole blood was immediately diluted 1:1 with PBS, layered over Ficoll, and centrifuged at 1,300 × *g* for 30 min to separate PBMCs. Cells were washed twice with PBS before being cultured at 10^6^ cells/mL in RPMI 1640 media supplemented with 1.77 mM l-glutamine, 76 μM streptomycin, 44 IU penicillin, 7.44 mM HEPES, and 8.9% hiFBS and containing 0, 10, 100, or 200 nM mercuric chloride (HgCl_2_) (with PBS as a vehicle). Cells were also separately cultured with the same concentrations of HgCl_2_ in the presence of 50 ng/mL LPS. Each treatment group was established in triplicate. PBMCs were maintained in culture for 48 hr at 37°C and 5% CO_2_. Cells were harvested by gentle agitation, followed by centrifugation for 5 min at 2,000 × *g*. Cell culture supernatants were stored in aliquots at −80°C until analysis.

### Cytokine measurement

Cell culture supernatants were thawed on ice and analyzed for cytokine content using the multiplex bead-based Bio-Plex suspension array for cytokines (Bio-Rad, Hercules, CA) according to the manufacturer’s instructions. The following seven cytokines were measured in all samples: interleukin-1β (IL-1β; detection range, 0.6–2,527 pg/mL), IL-1 receptor antagonist (IL-1Ra; 5.5–22,701 pg/mL), IL-4 (0.1–720 pg/mL), IL-10 (0.9–1,808 pg/mL), IL-17 (3.3–6,985 pg/mL), interferon-γ (IFN-γ; 6.4–20,882 pg/mL), and tumor necrosis factor-α (TNF-α; 1.6–55,716 pg/mL). For three subjects, we compared cytokine measurements for TNF-α obtained by the multiplex assay with measurements obtained using an enzyme-linked immunosorbent assay (Quantikine, R&D Systems, Minneapolis, MN) to validate the multiplex assay (correlation between assay measurements: *R*^2^ = 0.966; data not shown).

### Statistical analyses

We first plotted and examined data on a natural scale and then compared median cytokine concentrations for each Hg treatment group. The distribution of cytokine concentrations for each Hg treatment group was examined by calculating the interquartile range (IQR) of all observed responses. Because cytokine data are strongly right-skewed, the data were log-transformed using the natural logarithm. We used STATA10 software (version 10IC; StataCorp, College Station, TX) and R (version 2.8.0; R Foundation for Statistical Computing 2008) to generate graphs.

Two models—a simple linear regression (model 1) and a three-level hierarchical linear regression model with random intercept (model 2)—were then fit to the log-transformed data for each cytokine. Models were fit using Bayesian posterior inference based on Markov chain Monte Carlo simulation. We used WinBUGS software (version 1.4; MRC Biostatistics Unit, Cambridge, UK) to analyze the data, with noninformative prior distributions (Crainiceanu et al. 2005; Lunn et al. 2000). We used 100,000 iterations to estimate model parameters; the first 10,000 iterations were discarded. Posterior median estimates and 95% credibility intervals (CIs) are reported for parameters in each model.

Data from the phase 2 participants were modeled (model 3) using a linear mixed-effects model with two-level hierarchy for the random intercept. Model 3 had two levels instead of three because phase 2 volunteers had only one visit, whereas phase 1 volunteers had six visits. The posterior median estimates of model 3 were then compared with the estimates for the 95% CI of model 2 parameters to analyze the predictive properties of model 2 for phase 2 volunteer responses.

Finally, data from the phase 1 and phase 2 participants were combined and used in an exploratory analysis of the effects of both subject-level and visit-level characteristics on the intercept and slope of the concentration–response curve for TNF-α (model 6).

## Results

### Blood donor characteristics

The phase 1 data set consisted of complete concentration–response data for seven cytokines on 111 separate visits from 20 volunteers. One volunteer moved away after completing only five visits; one volunteer began taking birth control pills after three visits; and the remaining five visits were censored because of laboratory errors. Characteristics of all phase 1 and phase 2 participants are summarized in Table 1.

### LPS enhances HgCl_2_ modulation of cytokine release

The effects of HgCl_2_ treatment on cytokine release, both in the presence and in the absence of LPS, are summarized in Table 2 for all visits of all phase 1 volunteers. Changes in cytokine concentration were not due to nonspecific cytotoxic effects of Hg [see Supplemental Material, Figure 2 (doi:10.1289/ehp.0900855.S1)]. We also did not observe any changes in cell subpopulations (CD3^−^CD19^+^ B cells, CD3^+^CD4^+^ T cells, CD3^+^CD8^+^ T cells, CD11b^+^ monocytes and macrophages) as a result of HgCl_2_ treatment within PBMC cultures from a subset of six volunteers [see Supplemental Material, Table 1 (doi:10.1289/ehp.0900855.S1)].

In the presence of LPS, the IQRs observed for each cytokine were large, reflecting the large amount of variation seen in cytokine response at different visits and among different volunteers. As expected for nonstimulated and noncontaminated cultures, cytokine levels were low in the absence of LPS. The magnitude of the changes due to HgCl_2_ treatment observed in the presence of LPS was much greater compared with the changes observed in the absence of LPS. In this article we focus on the effects of HgCl_2_ treatment on cytokine release from cells that have been stimulated with LPS.

### Cytokine responses and model selection

We explored data on cytokine release in response to HgCl_2_ treatment using several models. To ensure that cytokine concentration data are normally distributed, we used log-transformed data to model effects of HgCl_2_ on cytokine release [see Supplemental Material, Figure 3 (doi:10.1289/ehp.0900855.S1)].

Model 1 is a simple linear regression:


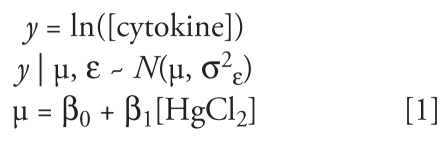


This model describes the mean cytokine response (μ) as a concentration–response curve consisting of an intercept β_0_, which describes the baseline cytokine response to LPS in the absence of HgCl_2_, and a slope β_1_. σ^2^_ε_ quantifies the observed variability that is unexplained by the model. This model does not take into account the correlated and hierarchical nature of the data set.

The variation observed in the intercept of the concentration–response curves suggests that a model with a random intercept may be more appropriate to describe the data set [see Supplemental Material, Figure 3 (doi:10.1289/ehp.0900855.S1)]. Model 2 is a multilevel linear regression model with a random intercept:


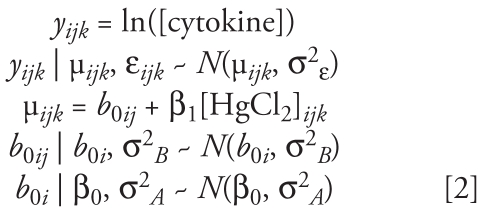


This model accounts for the fact that each observation, *k*, is made within a particular visit, *j*, from a particular volunteer, *i*. This model assumes that the observed cytokine response for the *i*th subject at the *j*th visit, *y_ijk_*, is normally distributed about its mean, μ*_ijk_*, with a variance, quantified by σ^2^_ε_, which again quantifies the observed variability that is unexplained by the model. Model 2 is similar to model 1 in that the mean cytokine response is modeled as a function of an intercept, β_0_, and a slope, β_1_, which describe the concentration–response curve. The variance about β_0_ has been further apportioned by the introduction of a random intercept for each visit, *b*_0_*_ij_*, and each subject, *b*_0_*_i_*. The random intercept for each visit is assumed to be normally distributed about the mean subject intercept, *b*_0_*_i_*, with variance σ^2^*_B_*, which quantifies the amount of within-individual variation observed within a subject *i* over *j* multiple visits. The mean subject random intercepts, *b*_0_*_i_*, are assumed to be normally distributed about the overall mean intercept of the data set, β_0_, with a variance σ^2^*_A_*, which quantifies the amount of between-individual variation observed.

**Figure 1:**
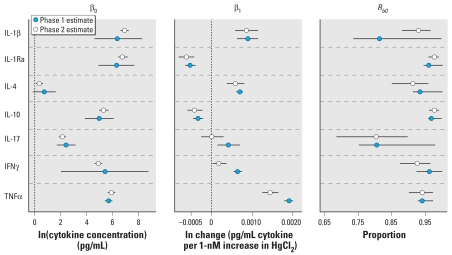
Model estimates [median (95% CI)] derived from phase 1 volunteers predict the response to LPS and HgCl_2_ in phase 2 volunteers. Model 2 estimates for concentration–response curves for each of the seven different cytokines derived from the log-transformed data from 111 visits of the 20 phase 1 volunteers are compared with model 3 estimates derived from the single visit of the 20 phase 2 volunteers. β_0_ is the estimate for the intercept of the HgCl_2_ concentration–response curve; β_1_ is the estimate for slope (log change in pg/mL of cytokine per 1-nM increase in HgCl_2_); and *R_b_*_0_ is the proportion of variation in the data set that is explained by variation in the baseline response to LPS. The vertical dashed lines for β_0_ and β_1_ indicate zero.

Parameter estimates for each model for each cytokine are summarized in Table 3. Model 2 estimates (derived from phase 1 volunteers) are depicted as solid blue circles in Figure 1. Supplemental Material, Figures 3–6 (doi:10.1289/ehp.0900855.S1), show close agreement between the observed cytokine data and model 2 predictions for each cytokine. Although the median estimates from both models for β_0_ and β_1_ are similar, model 2 estimates for β_1_ tend to be more precise, revealing a negative slope for IL-10 and IL-1Ra that is otherwise obscured. The estimates for σ^2^_ε_ are notably smaller for model 2 compared with model 1, suggesting that model 2 describes a larger portion of the variability in cytokine response.

HgCl_2_ treatment significantly increased the release of the proinflammatory cytokines TNF-α and IL-1β in a concentration–response manner, indicated by a positive value for the slope β_1_. HgCl_2_ treatment also caused a significant reduction in IL-1Ra and IL-10 release. We consistently observed each of these effects of HgCl_2_ on cytokine release both among subjects and within subjects over the course of six visits. In addition to affecting the balance of proinflammatory and antiinflammatory cytokines, HgCl_2_ also caused a significant increase in IFN-γ, IL-4, and IL-17.

To compare the slopes of the concentration–response curves between the cytokines, we calculated the coefficient of variation (CV) for each cytokine. The CV measures the noise in the data compared with the signal by dividing the SD by the mean for each β_1_ value. TNF-α has the lowest CV value (3%), indicating that TNF-α showed the largest and most consistent changes in response to HgCl_2_ treatment compared with the other cytokines (IL-4, 6%; IFN-γ, 8%; IL-1Ra, 12%; IL-1β, 15%; IL-10, 18%; IL-17, 33%).

Estimates for the between-subject variation, σ^2^*_A_*, and the within-subject variation, σ^2^*_B_*, are also shown in Table 3. σ^2^*_B_* accounts for a relatively large proportion (> 90% of the overall variation for IL-1β, IL-1Ra, IL-4, and TNF-α) of the overall variation in the data set described by σ^2^_ε_ in model 1. σ^2^*_A_* is not precisely estimated from these data for any cytokine, suggesting that a larger sample size would have been necessary. We simulated a larger data set with similar effect size and variance to estimate the necessary sample size. Recruiting 45 volunteers with six visits each would be adequate to precisely estimate σ^2^*_A_*, although recruiting 50 volunteers with three visits each would be a more efficient approach to attain the necessary power [see Supplemental Material, Figure 7 (doi:10.1289/ehp.0900855.S1)].

### Comparison of phase 1 and phase 2 model parameters

To validate the predictive capability of these models, we cultured PBMCs from 20 new subjects (phase 2). After the same *in vitro* treatments with HgCl_2_ and LPS as used in phase 1, we measured cytokine release for the same seven cytokines.

The log-transformed cytokine response data (*k*) were fit with model 3:


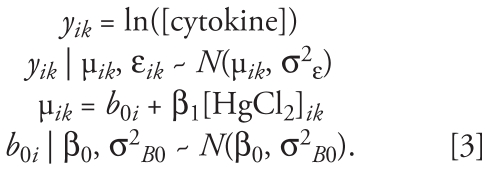


We compared median estimates and 95% CIs for β_0_ and β_1_ obtained from model 3 using data from 20 phase 2 subjects with estimates obtained for each cytokine by fitting data from the 20 phase 1 subjects with model 2 (Figure 1). We hypothesized that median estimates from phase 2 would fall within the 95% CI for each model parameter predicted based on phase 1 data. Overall, the phase 1 model accurately predicts the magnitude and direction of the slope β_1_ of the concentration–response curve for each cytokine, along with accurately predicting the range of the intercept β_0_.

Multilevel mixed effects models provide a natural decomposition of observed variance into within- and between-subject variance and noise variance. A measure of the proportion of total variance in model 2 explained by within- and between-subject variation is *R_b_*_0_:


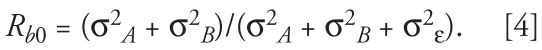


For model 3, *R_b_*_0_ is somewhat simpler, represented as the proportion of variance explained by the random intercept compared with the total variance:


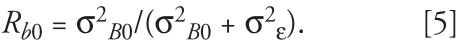


The *R_b_*_0_ statistic computed for model 3 was also accurately predicted by model 2, based on data from phase 1 subjects. Estimates for *R_b_*_0_ for models 2 and 3 are shown in Figure 1.

### Subject-specific characteristics may contribute to the variation observed in cytokine response

Although our sample size is too small to generate an in-depth, multivariate exploration of the effects of subject- and visit-specific characteristics on cytokine response, we were able to use the questionnaire data collected on health and lifestyle in a hypothesis-generating, exploratory analysis of the effects of these characteristics on TNF-α release. We combined the data sets for both phase 1 and phase 2 participants for this analysis:


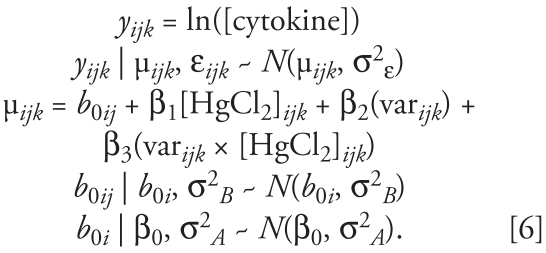


**Figure 2:**
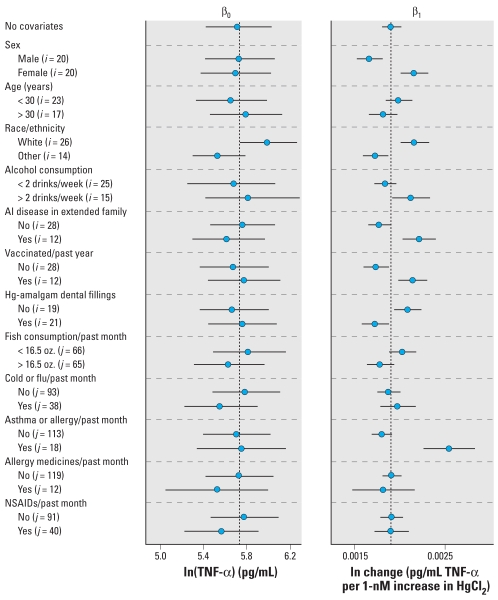
Subject- and visit-specific variables may affect response to LPS and HgCl_2_. AI, autoimmune. Total data for TNF-α release for all subjects (*i* = 40) and all visits (*j* = 131) were evaluated with model 6. The effect of each covariate was tested on both the intercept (β_0_) and the slope (β_1_) using concentration–response data from the specified subpopulations (see Table 1 for covariate descriptions). The estimated median and 95% CI for each model parameter were compared with the median model estimates from a model with no covariates (model 2), represented by the dashed vertical lines.

In addition to the multilevel random intercept, model 6 contains the terms “[HgCl_2_]*_ijk_*” and “var*_ijk_*” as explanatory variables. “Var” represents each variable (e.g., sex) that was tested individually in the model. Potential interaction between each variable and HgCl_2_ treatment was modeled with the term var*_ijk_* × [HgCl_2_]*_ijk_*. Posterior distributions were computed for the intercept (β_0_ + β_2_) and slope (β_1_ + β_3_) for each subpopulation examined (e.g., males and females). These estimates are compared with the estimates for the intercept (β_0_) and slope (β_1_) of the TNF-α concentration–response curve derived from model 2 using data from all 40 subjects (Figure 2).

None of the variables tested had a statistically significant impact on the estimate for the intercept. Ethnicity had the largest effect on the intercept, with nonwhite participants showing a tendency toward a higher value. Having an allergic reaction or asthma attack had the largest impact on the estimate for β_1_. Being female or nonwhite, consuming more than two alcoholic beverages per week, having an extended family member with autoimmune disease, being vaccinated within 1 year of the visit, and having no dental fillings made of Hg amalgam all also increased the estimate of β_1_ compared with model 2 estimates. These results suggest that individual-level characteristics, as well as visit-level characteristics, may affect the intercept and slope of the concentration–response curve for TNF-α.

## Discussion

In this study we found that low, physiologically relevant concentrations of iHg up-regulate the release of proinflammatory TNF-α and IL-1β and down-regulate antiinflammatory IL-10 and IL-1Ra release in a concentration–response fashion in LPS-stimulated human PBMCs *in vitro*. The highest HgCl_2_ concentration we used (200 nM) corresponds to a blood Hg level of 37 μg/L, which has been observed in populations exposed to Hg occupationally or through consumption of methylmercury-contaminated fish and is well within the range of U.S. exposures (Crompton et al. 2002; Hightower and Moore 2003). LPS used to stimulate the PBMCs in this system interacts with the Toll-like receptor 4 (TLR4) receptor complex on monocytes and macrophages. Exposure to infectious agents that stimulate TLR4 signaling is known to modulate the risk of autoimmune disease in humans (Cooke et al. 2008) and is critical to some animal models of autoimmunity (Frisancho-Kiss et al. 2007). A growing literature indicates that Hg can interact with infectious disease stimuli to increase inflammation and exacerbate autoimmune disease (Abedi-Valugerdi et al. 2005; Silbergeld et al. 2005). Here we have provided further evidence that Hg can interact with infectious disease stimuli, in this case modulating the cytokine response to LPS.

Regulation of proinflammatory cytokines is tightly controlled in physiologically appropriate immune responses. Induction of antiinflammatory responses generally occurs soon after or concurrently with proinflammatory cytokine induction (Arend 2002). Imbalance in pro- and antiinflammatory cytokine production has been implicated in the etiology of many diseases, including autoimmune diseases and atherosclerosis (Apostolakis et al. 2008; Suh and Kim 2008). Our model predicts an increase of 119 pg/mL IL-1β and 141 pg/mL TNF-α over a 200 nM increase of HgCl_2_, which well reflects the observed increases in our study. Changes of this magnitude are biologically significant, because lupus patients have elevated serum TNF-α and IL-1β concentrations of a similar magnitude compared with controls (Sabry et al. 2006; Suh and Kim 2008). The Hg-induced increases in proinflammatory cytokine release are unopposed by the physiologically appropriate antiinflammatory response; in fact, IL-1Ra and IL-10 release are suppressed by Hg treatment.

Most rodent studies of Hg-induced autoimmunity have focused on the influence of T_H_1 and T_H_2 cell populations (Hu et al. 1999). Here we show evidence that in humans T_H_1 and T_H_2 subsets may also be affected by Hg because both IL-4 and IFN-γ are slightly up-regulated in response to iHg treatment in activated PBMCs. Because other cell populations can produce these cytokines—and we observe both up-regulation of IL-4 and down-regulation of IL-10, which can both be produced by T_H_2 cells—further study is necessary to ascertain the cell populations affected by iHg. IL-17 is also up-regulated in response to Hg treatment. IL-17 is produced by CD4^+^ T_H_17 cells, which play a role in the etiology and pathology of autoimmune disorders (Wilson et al. 2007).

In the data presented here, the variability in the slope of the iHg concentration–response curve is relatively small compared with the variability observed in baseline response to LPS, indicating that the baseline response determined the relative cytokine release in the presence of iHg (i.e., participants who had a strong inflammatory response to LPS alone had much higher absolute levels of proinflammatory cytokines after Hg exposure *in vitro*). The apparent influence of variability observed in the baseline inflammatory response could have implications for variations in *in vivo* susceptibility to the immunotoxic effects of Hg. Those individuals who mount a more intense inflammatory response in the presence of an infectious stimulus may be at greater risk of pathology due to increased inflammation as a result of Hg exposure. We speculate that individuals with genetic susceptibility to diseases characterized by dysregulation of inflammation may be at particular risk of pathophysiologic consequences as a result of Hg exposure. An emerging emphasis on the inflammatory basis of cardiovascular disease is particularly intriguing (Apostolakis et al. 2008; Boyle 2005), given that a significant association has been observed between elevated methylmercury exposures resulting from fish consumption and increased risk for myocardial infarction despite the cardioprotective effects of fish consumption (Guallar et al. 2002). Our results further support the need to investigate interactions among Hg exposure, inflammation, and chronic disease risk.

Human PBMCs are receiving increasing attention, especially in the area of regulatory toxicology, and are a popular choice as a model system in immunotoxicology (Chaudhary et al. 2004; Giese et al. 2006; Hoffmann et al. 2005). The use of PBMCs fulfills the vision recently set forth by the U.S. National Research Council for the future of toxicology testing, in which human cells are studied *in vitro* for xenobiotic effects on signaling pathways (National Research Council 2007). However, the large amount of variability that is observed in response to xenobiotics in PBMCs taken from different individuals presents an analytical challenge when interpreting data from this model system. The variability observed *in vitro* is not an artifact of the model but reflects the highly variable nature of the human immune response (Coury et al. 2008; Garner-Spitzer et al. 2008; Love et al. 2008). As such, the use of human PBMCs in culture represents an opportunity to study and quantify the range of human responses to xenobiotics. This information has the potential to be of value during the process of risk assessment because uncertainty factors of 3 or 10 are normally applied in the process of defining acceptable exposure levels in order to account for variability in human responses (National Research Council 1983). Using a quantitative statistical method such as we have used in this study could allow for informative and appropriate factors to be applied in this process, based on the level of variability observed within the assay end points in a variety of model systems.

## Conclusions

Low concentrations of iHg affect immune function in human cells by dysregulation of cytokine signaling pathways. Given the integral nature of cytokine signaling in all aspects of immune function, these effects may have the potential to influence diverse health outcomes such as susceptibility to infectious disease or risk of autoimmunity. The present study demonstrates the importance of considering the immune system as a specific target of Hg toxicity and thus indicates the need for continued study of the role of Hg exposure in infections and chronic disease.

## Figures and Tables

**Table 1:** Population characteristics.

Variable	Phase 1 volunteers^a^	Phase 2 volunteers^b^
Subject-level variables		
Age (years)		
Mean ± SD	28.4 ± 4.99	27.7 ± 4.7
Range	22–39	22–39
Sex [no. (%)]		
Male	10 (50)	10 (50)
Female	10 (50)	10 (50)
Ethnicity [no. (%)]		
White	11 (55)	15 (75)
Black	4 (20)	2 (10)
Asian American	5 (25)	3 (15)
Extended family history of autoimmune disease [no. (%)]		
Yes	6 (30)	6 (30)
No	14 (70)	14 (70)
Vaccinated within 1 year [no. (%)]		
Yes	9 (45)	3 (15)
No	11 (55)	17 (85)
Smoking habits [no. (%)]		
Some days	1 (5)	3 (15)
Not at all	19 (95)	17 (85)
No. of alcoholic beverages/week		
Mean ± SD	3.0 ± 3.5	3.7 ± 2.9
Range	0–15	0–10
No. of dental fillings		
Mean ± SD	2.8 ± 3.9	1.7 ± 2.4
Range	0–12	0–8

Visit-level variables		
Dental work in past month [no. (%)]		
Yes	11 (9.9)	1 (5)
No	100 (90.1)	19 (95)
Total fish consumption in past month (oz)		
Mean ± SD	21.7 ± 22.8	24.2 ± 17.8
Range	0–134	0–60
Predatory fish consumption in past month (oz)		
Mean ± SD	8.2 ± 12.4	10.7 ± 15.3
Range	0–90	0–56
Nonpredatory fish consumption in past month (oz)		
Mean ± SD	13.1 ± 15.8	13.6 ± 10.7
Range	0–84	0–32
Date of visit [no. (%)]		
April–September	65 (59)	12 (60)
October–March	46 (41)	8 (40)
Used prescription medicines within 1 month [no. (%)]		
Yes	26 (23.4)	4 (20)
No	85 (76.6)	16 (80)
Used NSAIDs within 1 month [no. (%)]		
Yes	38 (34.2)	2 (10)
No	73 (65.8)	18 (90)
Used allergy medicines within 1 month [no. (%)]		
Yes	10 (9.0)	1 (5)
No	101 (91.0)	19 (95)
Reported cold or flu within 1 month [no. (%)]		
Yes	28 (25.3)	9 (45)
No	83 (74.7)	11 (55)
Reported asthma or allergy within 1 month [no. (%)]		
Yes	14 (12.6)	3 (15)
No	97 (87.4)	17 (85)

a Phase 1 volunteers (*i* = 20) were asked to donate blood six times for a total of 111 visits (after dropout and laboratory errors).

b Phase 2 volunteers (*i* = 20) were asked to donate blood at a single visit.

**Table 2:** Cytokine response summary.

		HgCl_2_ treatment	
Cytokine	LPS	0 nM	200 nM
IL-1β	Negative	2.0 (0.95–4.5)	2.7 (0.9–5.3)
	Positive	525.8 (372.7–851.9)	617.1 (418.8–1255.3)

IL-1Ra	Negative	21.4 (10.9–58.2)	24.8 (12.8–63.3)
	Positive	527.3 (270.3–985.0)	473.6 (266.9–880.5)

IL-4	Negative	0.3 (0.1–0.4)	0.3 (0.1–0.4)
	Positive	2.0 (1.6–2.8)	2.3 (1.8–3.3)

IL-10	Negative	1.6 (1.1–2.5)	1.3 (0.9–2.2)
	Positive	143.6 (85.4–266.4)	130.8 (75.0–277.4)

IL-17	Negative	8.8 (4.5–11.9)	8.1 (4.1–11.2)
	Positive	14.5 (11.1–18.2)	14.8 (11.2–19.8)

IFN-γ	Negative	14.5 (7.0–31.2)	14.1 (7.1–30.9)
	Positive	216.5 (152.3–363.7)	248.1 (174.8–381.8)

TNF-α	Negative	3.7 (2.6–5.0)	4.5 (2.8–6.8)
	Positive	338.9 (181.3–471.4)	456 (258.6–710.7)

Values shown are median (IQR) in pg/mL and include all visits from all phase 1 volunteers.

**Table 3:** Model parameter estimates (95% CIs).

Cytokine	Model	β_0_	β_1_	σ^2^*_A_*	σ^2^*_B_*	σ^2^ɛ
IL-1β	1	6.35 (6.29–6.41)	8.68 × 10^−4^ (3.17 × 10^−4^ to 14.3 × 10^−4^)	—	—	0.495 (0.453–0.542)
	2	6.41 (4.61–8.27)	8.97 × 10^−4^ (6.38 × 10^−4^ to 11.5 × 10^−4^)	0.0898 (0.0018−43.1)	0.361 (0.267–0.505)	0.109 (0.0990–0.119)

IL-1Ra	1	6.31 (6.24–6.38)	5.39 × 10^−4^ (−11.5 × 10^−4^ to 6.47 × 10^−4^)	—	—	0.781 (0.723–0.845)
	2	6.32 (4.94–7.66)	−5.25 × 10^−4^ (−6.54 × 10^−4^ to −3.99 × 10^−4^)	0.0413 (9.64 × 10^−4^ to 22.74)	0.742 (0.570–0.987)	0.0347 (0.0320–0.0378)

IL-4	1	0.738 (0.704–0.773)	6.09 × 10^−4^ (3.80 × 10^−4^ to 9.99 × 10^−4^)	—	—	0.204 (0.189–0.221)
	2	0.742 (–0.123 to 1.594)	7.05 × 10^−4^ (6.20 × 10^−4^ to 7.88 × 10^−4^)	0.0185 (8.20 × 10^−4^ to 9.25)	0.187 (0.144–0.250)	0.0153 (0.0141–0.0166)

IL-10	1	4.97 (4.90–5.04)	−3.86 × 10^−4^ (−10.2 × 10^−4^ to 2.44 × 10^−4^)	—	—	0.850 (0.787–0.920)
	2	4.97 (3.86–6.07)	−3.33 × 10^−4^ (−4.50 × 10^−4^ to −2.18 × 10^−4^)	0.0255 (7.86 × 10^−4^ to 14.9)	0.842 (0.647–1.12)	0.0291 (0.0269–0.0317)

IL-17	1	2.45 (2.39–2.52)	4.01 × 10^−4^ (−2.04 × 10^−4^ to 10.0 × 10^−4^)	—	—	0.773 (0.716–0.837)
	2	2.45 (1.71–3.17)	4.27 × 10^−4^ (1.51 × 10^−4^ to 6.98 × 10^−4^)	0.0122 (6.46 × 10^−4^ to 6.49)	0.623 (0.477–0.834)	0.160 (0.148–0.174)

IFN-γ	1	5.45 (5.40–5.49)	6.31 × 10^−4^ (2.22 × 10^−4^ to 10.4 × 10^−4^)	—	—	0.375 (0.347–0.405)
	2	5.42 (2.04–8.81)	6.56 × 10^−4^ (5.53 × 10^−4^ to 7.61 × 10^−4^)	0.329 (0.0253−131.32)	0.276 (0.214–0.367)	0.0240 (0.0221–0.0260)

TNF-α	1	5.67 (5.61–5.72)	20.0 × 10^−4^ (15.5 × 10^−4^ to 25.0 × 10^−4^)	—	—	0.501 (0.464–0.541)
	2	5.66 (4.13–7.21)	20.0 × 10^−4^ (18.8 × 10^−4^ to 21.1 × 10^−4^)	0.0592 (0.0014−28.9)	0.459 (0.355–0.608)	0.0308 (0.0285–0.0334)

Model estimates are derived from log-transformed data from phase 1 volunteers for cells treated with LPS. Model 1 is a simple linear regression, and model 2 is a three-level hierarchical linear mixed-effects model.
